# Mg/ZrO_2_ Metal Matrix Nanocomposites Fabricated by Friction Stir Processing: Microstructure, Mechanical Properties, and Corrosion Behavior

**DOI:** 10.3389/fbioe.2021.605171

**Published:** 2021-03-25

**Authors:** Ke Qiao, Ting Zhang, Kuaishe Wang, Shengnan Yuan, Shengyi Zhang, Liqiang Wang, Zhi Wang, Pai Peng, Jun Cai, Chaozong Liu, Wen Wang

**Affiliations:** ^1^School of Metallurgical Engineering, National and Local Joint Engineering Research Center for Functional Materials Processing, Xi’an University of Architecture and Technology, Xi’an, China; ^2^State Key Laboratory of Metal Matrix Composites, School of Materials Science and Engineering, Shanghai Jiao Tong University, Shanghai, China; ^3^Institute of Orthopaedics and Musculoskeletal Science, University College London, Royal National Orthopaedic Hospital, Stanmore, United Kingdom

**Keywords:** Friction stir processing, microstructure, mechanical properties, corrosion properties, texture

## Abstract

Magnesium (Mg) and its alloys have attached more and more attention because of their potential as a new type of biodegradable metal materials. In this work, AZ31/ZrO_2_ nanocomposites with good uniformity were prepared successfully by friction stir processing (FSP). The scanning electron microscope (SEM) and transmission electron microscope (TEM) were used to characterize the microstructure of the composites. The mechanical properties, electrochemical corrosion properties and biological properties were evaluated. In addition, the effect of reinforced particles (ZrO_2_) on the microstructure and properties of the composite was studied comparing with FSP AZ31 Mg alloy. The results show that compared with the base metal (BM), the AZ31/ZrO_2_ composite material achieves homogenization, densification, and grain refinement after FSP. The combination of dynamic recrystallization and ZrO_2_ particles leads to grain refinement of Mg alloy, and the average grain size of AZ31/ZrO_2_ composites is 3.2 μm. After FSP, the c-axis of grain is deflected under the compression stress of shoulder and the shear stress of pin. The ultimate tensile strength (UTS) and yield strength (YS) of BM were 283 and 137 MPa, respectively, the UTS and YS of AZ31/ZrO_2_ composites were 427 and 217 MPa, respectively. The grain refinement and Orowan strengthening are the major strengthening mechanisms. Moreover, the corrosion resistance in simulated body fluid of Mg alloy is improved by grain refinement and the barrier effect of ZrO_2_.

## Introduction

Magnesium (Mg) and its alloys are considered to have great potential in biomedical application due to the high strength-to-weight ratio, good biocompatibility and promotion of bone cell healing (Staiger et al., 2006; Castellani et al., 2011; Henderson et al., 2014). However, the high corrosion and degradation rates *in vivo*, and the low strength limit their application development (Morisada et al., 2006a). Previous studies have shown that the Mg matrix composites prepared by adding secondary particles to Mg can significantly reduce the corrosion and degradation rates, thus improve strength, corrosion resistance, and biocompatibility (Lan et al., 2004; Wang et al., 2004; Ugandhar et al., 2006).

At present, powder metallurgy (Davis and Ward, 1993), in-situ fabrication (Daniel et al., 1997), spray deposition (Lavernia and Grant, 1988), and stir casting (Hashim et al., 1999) are commonly used to fabricate composites. However, the composites fabricated by the above methods have many defects such as voids, which is eliminated by severe plastic deformation methods such as extrusion and rolling, leading to a longer process and higher cost. Therefore, it is necessary to develop an effective technique to prepare high-quality composites.

Friction stir processing (FSP) is an alternative solid-state processing technology for producing Mg matrix composites, based on the principles of friction stir welding (FSW), potentially addressing the above-mentioned limitations (Mishra and Ma, 2005). Specifically, FSP can achieve the homogenization, densification, and grain refinement of microstructure simultaneously (Ammouri et al., 2015; Ni et al., 2016; Xu and Bao, 2016), thus improve the mechanical properties and corrosion properties of materials. In 2006, Morisada et al. (2006b) first prepared AZ31/MWCNTs (multi-walled carbon nanotubes) surface composites by FSP. It was reported that the low temperature could avoid the interfacial reaction between reinforced particles and Mg matrix during FSP. Furthermore, severe plastic deformation contributed to the fragment and uniform mixing of reinforced particles. This work has attracted many research interests in the preparation of Mg matrix composites by FSP.

So far, a lot of reinforced particles, such as TiC (Balakrishnan et al., 2015; Navazani and Dehghani, 2015), TiAlC (Gobara et al., 2015), Al_2_O_3_ (Azizieh et al., 2018), B_4_C (Vedabouriswaran and Aravindan, 2018), MWCNT (Morisada et al., 2006b; Arab et al., 2017), and SiC (Morisada et al., 2006a) have been added in Mg matrix by FSP, improving the mechanical properties of the alloys. For example, Navazani and Dehghani (2015) and Balakrishnan et al. (2015) studied the microstructure and mechanical properties of AZ31/TiC composites fabricated by FSP. The results showed that TiC particles distributed uniformly in AZ31 Mg matrix after FSP, which promoted the grain refinement and improved the microhardness. According to the research of Azizieh et al. (2018), adding Al_2_O_3_ to AZ31 Mg alloy with FSP effectively increased the wear resistance of the material. It has been reported (Vedabouriswaran and Aravindan, 2018) that after adding B_4_C particles to RZ 5 Mg alloy, the coarse grains in base metal (BM) become the fine grains in composites due to the pinning effect of reinforced particles. The microhardness and tensile strength from 81 HV and 200 MPa (BM) increased to 403 HV and 320 MPa (composites), respectively. This phenomenon also be founded in AZ31/SiC composites fabricated by FSP (Morisada et al., 2006a). However, there are few reports on the above-mentioned reinforcing particles for improving biocompatibility of Mg alloys, it is vital to find other materials to address the above problem.

Studies have shown that the addition of ZrO_2_ particles in Mg matrix contributes to the mechanical properties and biocompatibility of materials (Navazani and Dehghani, 2016; Vignesh et al., 2019). For example, Navazani and Dehghani (2016) added ZrO_2_ particles to AZ31 Mg plate through FSP, then observed that the particles promote the grain refinement and improve the mechanical properties of composites. Vignesh et al. (2019) prepared AZ91D-ZrO_2_ surface composites by FSP. It was reported that the combination of FSP and ZrO_2_ reduced the grain size, and broke and disperse the secondary particles. The dispersion of ZrO_2_ particles can increase the accumulated surface potential, thereby improving the corrosion resistance of composites. However, only simply characterize and evaluate the microstructure and performance of ZrO_2_/Mg matrix composites in previous researches, the influence mechanism of microstructure on mechanical properties and corrosion resistance has not been thoroughly and comprehensively analyzed and discussed.

In view of the above problems, AZ31/ZrO_2_ nanocomposites are prepared in this work by FSP. The microstructure, mechanical properties, and corrosion resistance of AZ31 Mg alloy and AZ31/ZrO_2_ nanocomposites are analyzed in detail to clary the influence of FSP and ZrO_2_ particles on the Mg alloy, respectively. The present work intends to provide a new insight for the preparation of biomedical Mg matrix composites.

## Materials and Experimental Methods

### Materials Preparation

**FIGURE 1:**
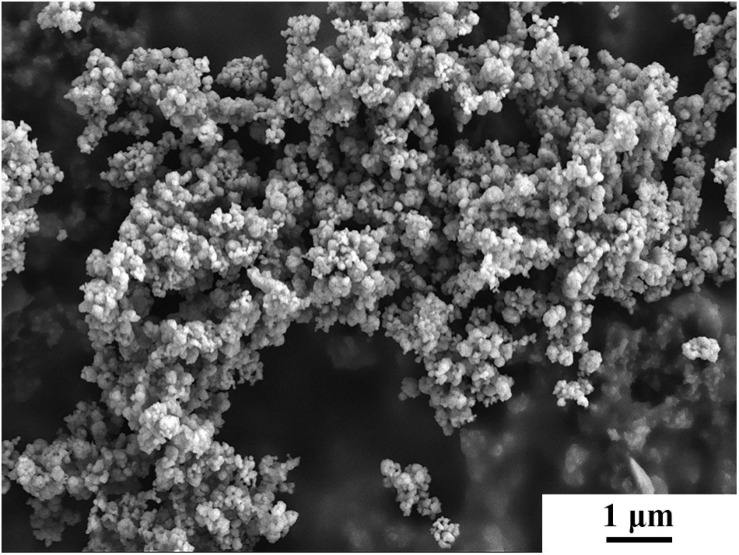
SEM micrograph of ZrO_2_ powder.

**FIGURE 2:**
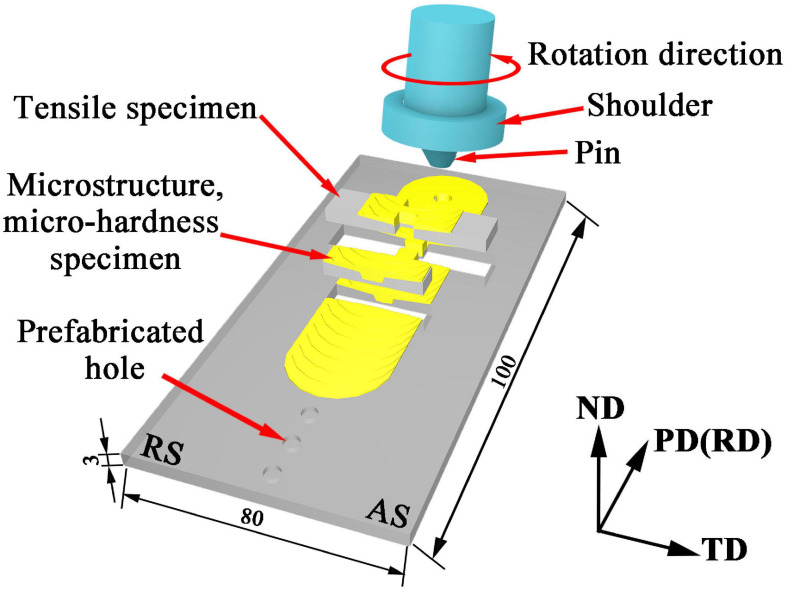
Schematic of the FSP. (AS and RS represent the advancing and retreating sides, respectively. PD, TD, and ND represent processing, transverse, and normal directions, respectively. Unit: mm).

A rolled AZ31 Mg plate with a dimension of 100 mm × 80 mm × 3 mm was used as Base metal (BM) in this work. ZrO_2_ powder with a diameter ranging from 50 to 150 nm and an average diameter of 80 nm was used as reinforcement as shown in Figure 1. The volume fraction of the ZrO_2_ particles added to the plate is about 17.6%. Holes with diameter of 3 mm, depth of 1 mm and hole spacing of 10 mm were drilled by a drilling machine on the surface of the AZ31 Mg alloy plates. After filling the prefabricated holes with ZrO_2_ particles, these plates were processed by FSP. The AZ31 Mg alloy plates with and without ZrO_2_ particles were processed on a FSP machine (LM-BM16), respectively. The stir tool consisting of cylindrical shoulder of 20 mm in diameter, pin of 2 mm in length and 4 mm in diameter. The tool rotation speed was 1180 rpm, the processing speed was 23.5 mm/min, the tilt angle was 2° and the plunge depth was 0.5 mm. All samples were processed six passes. The schematic of the FSP is shown in Figure 2. Here into, AZ31 Mg alloy sample without ZrO_2_ particles by FSP is marked as FSP, and the AZ31 Mg alloy sample with ZrO_2_ particles is marked as FSP-ZrO_2_.

### Microstructural Characterization

Metallographic specimens were taken along the perpendicular PD with a dimension of 20 mm × 5 mm × 3 mm, which were grinded, polished, and etched by picric acid etching solution (10 mL acetic acid + 10 mL water + 4.2 g picric acid dissolved in 100 mL alcohol) for 10 s. The microstructures were observed using scanning electron microscope (SEM, Gemini SEM 300) with electron backscatter diffraction (EBSD) at a voltage of ∼5 kv. The samples were electrolytically polished in a 10 vol.% of perchloric acid solution at a voltage of ∼10 V and a temperature of −20°C. TEM samples with a dimension of 10 mm × 10 mm × 1 mm were cut from stir zone (SZ) in FSP-ZrO_2_, and ground to a thickness of approximately to 40 μm with sandpaper, and then the twin-jet electropolishing was conducted using 6% perchloric acid ethanol solution at −30°C. TEM observations were performed on JEM-200CX equipment at a voltage of ∼120 kv.

### Mechanical Properties

The hardness sample with the dimension of 20 mm × 5 mm × 3 mm were cut along the vertical PD. The hardness testing was carried on the TD × ND plane using a 401MVD microhardness tester with a loading of 100 g and a dwell time of 10 s. Indentations spacing were 0.5 mm in this work. The tensile specimens with a gauge section dimension of 34 mm × 8 mm × 3 mm were cut along PD, ground and polished, then tested on an Instron 8,801 equipment at room temperature. The strain rate was 1.0 × 10^–3^ s^–1^. Each testing was repeated at least three times to ensure the accuracy of data. The fracture surfaces of tensile specimens were characterized by SEM.

### Electrochemical Corrosion Performance

The electrochemical corrosion samples with a dimension of 8 mm × 8 mm × 3 mm were cut from BM, FSP (SZ), and FSP-ZrO_2_ (SZ). After grinding and polishing the sample, the electrochemical test was performed on the Gamry Reference 600 + instrument. The corrosion solution was simulated body fluid (8.035 g/L). NaCl, 0.355 g/L NaHCO_3_, 0.225 g/L KCl, 0.231 g/L K_2_HPO_4_⋅3H_2_O, 0.311 g/L MgCl_2_⋅6H_2_O, 39 mL/L HCl (1 mol/L), 0.292 g/L CaCl_2_, 0.072 g/L NaSO_4_, 6.118 g/L Tris, 1 mol/L HCl) (Kokubo and Takadama, 2006). The samples were used as the working electrodes, Ag/AgCl (saturated KCl) and platinum strip were used as the reference and counter electrodes, respectively. Before recording the potentiodynamic polarization curves, the samples were soaked in the simulated body fluid (SBF) solution for 300 s to get the steady reaction condition. The impedance measurement scan frequency ranges from 100,000 Hz to 0.1 Hz with an excitation signal amplitude is 10 mV. The impedance data were analyzed using the ZSimpWin software. The initial potential is reduced by 500 mV relative to the open circuit potential (OCP), and the termination potential is increased by 1.5 V relative to the OCP. The scan rate is 1 mV/s, and the test temperature is room temperature.

### Scanning Vibrating Electrode Technique (SVET) Measurement

The SVET samples were cut from BM, and SZ of the FSP and FSP-ZrO_2_ samples with a dimension of 8 μm × 8 μm. After grinding and polishing, they were tested on the Princeton VersaSCAN instrument. The measurements were performed in SBF and at an OCP. The scanning range was 600 μm × 600 μm, the scanning speed was 10 μm/s, and the process combined surface scanning and line scanning. The SVET samples were immersed for 1, 3, 6, and 24 h, respectively. The statistical analysis of SVET data was carried out with Origin software. The voltages were displayed in a three-dimensional (3D) maps, which showed the spatial distribution of the voltage as a function of the (x, y) position in the scan region. The voltage value in the SVET map is positive for anodic currents and negative for cathodic currents. The contour map of the voltage was located at the bottom of the 3D map.

## Results

### Microstructure Evolution

**FIGURE 3:**
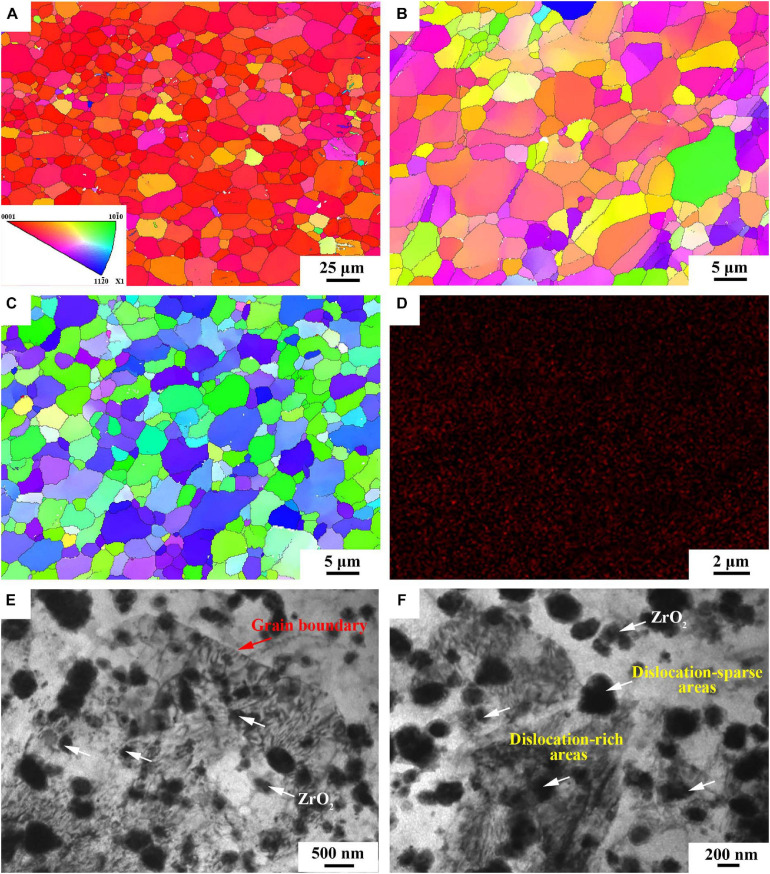
The IPF map of **(A)** BM, **(B)** FSP and **(C)** FSP-ZrO_2_; **(D)** The EDS map of FSP-ZrO_2_ sample; **(E)** and **(F)** TEM images of FSP-ZrO_2_ sample, respectively.

Figures 3A–C show the inverse pole figure (IPF) of BM, FSP and FSP-ZrO_2_ samples. BM exhibits equiaxed grains with the size ranging from 0.2 to 53.5 μm, and the average size is 10 μm (Figure 3A). Compared with BM, FSP displays a more uniform microstructure and finer grain, with a grain size range of 0.1–15 μm and an average size of 4.0 μm (Figure 3B). The microstructure of FSP-ZrO_2_ sample is further homogenized and refined, with a grain size range of 0.1–14 μm and an average grain size of 3.2 μm (Figure 3C), which indicates that ZrO_2_ particles contribute to reducing grain size. Morisada et al. (2006a) showed that FSP with the SiC particles can refine grains more effectively due to the enhancement of the induced strain and the pinning effect by the SiC particles. Therefore, it can be considered that the ZrO_2_ particles in this work play a similar role to the SiC particles in the report. Figure 3D shows the distribution of Zr elements in FSP-ZrO_2_ sample. Zr element is uniformly distributed in the Mg matrix without obvious vacancies and aggregation, indicating that the ZrO_2_ particles are evenly distributed in the composites after FSP. Figures 3E,F are TEM images of FSP-ZrO_2_ samples. It can be clearly seen that the ZrO_2_ particles (white arrows) are well combined with the matrix, and mainly nucleate within the grains rather than preferentially distribute along the grain boundaries (red arrows). In addition, due to the high temperature and severe plastic deformation during FSP, the dislocation around the ZrO_2_ particles is unevenly distributed, forming dislocation-rich areas (i.e., dislocation tangle) and dislocation-sparse areas (Figure 3F). During FSP, the stress is concentrated around the ZrO_2_ particles due to the significant difference between the elastic modulus of the ZrO_2_ particles and Mg matrix, thus a large number of dislocations are generated around ZrO_2_ particles. On the other hand, the dynamic recovery is reduced owing to the low stacking fault energy of Mg alloys, resulting in dense and sparse dislocations (Vignesh et al., 2019).

**FIGURE 4:**
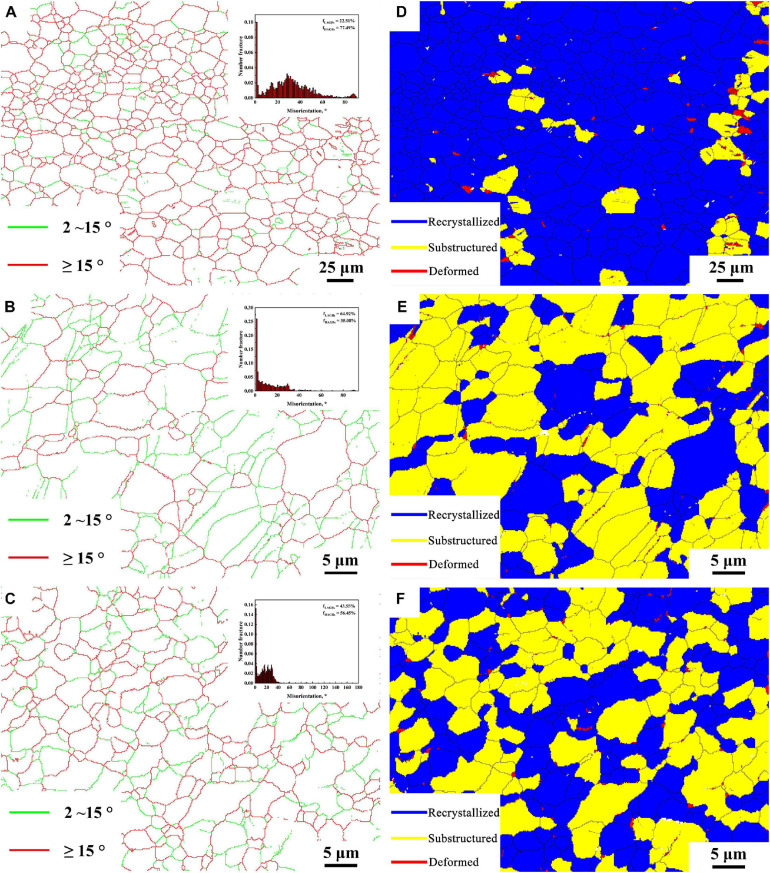
The grain boundary map: **(A)** BM, **(B)** FSP, **(C)** FSP-ZrO_2_; and recrystallized grain distribution map: **(D)** BM, **(E)** FSP, **(F)** FSP-ZrO_2_.

Figures 4A–F show the grain boundary distribution maps and recrystallized grain distribution maps of BM, FSP, and FSP-ZrO_2_ samples, respectively. The insets are the misorientation angle distribution maps. The green and red lines represent low angle grain boundaries (LAGBs) of 2∼15°and high angle grain boundaries (HAGBs) of >15°, respectively. Blue indicates recrystallized grains, yellow indicates sub-grains, and red indicates deformed grains. It can be seen that the proportions of HGABs in BM, FSP, and FSP-ZrO_2_ samples are 86.3, 52.8, and 66.5%, respectively. The proportions of recrystallized grains are 89.83, 34.37, and 48.79%, respectively. The HAGB ratios of the above three samples are all reduced because of the dislocations (Figures 3E,F) produced inside the grains during FSP. It is worth noting that there are a large number of 60°± 5° and 86° ± 5° twins in BM sample, 60° ± 5° twins in FSP sample are reduced, and two twins in FSP-ZrO_2_ composites disappear, as shown in the inserts in Figures 4A–C. The grain size affects twin deformation, the finer the grain, the more difficult to twin. Therefore, the main reason for the reduction of twins is that FSP and the combination of FSP with ZrO_2_ particles makes the grain refined (Figures 3B,C).

**FIGURE 5:**
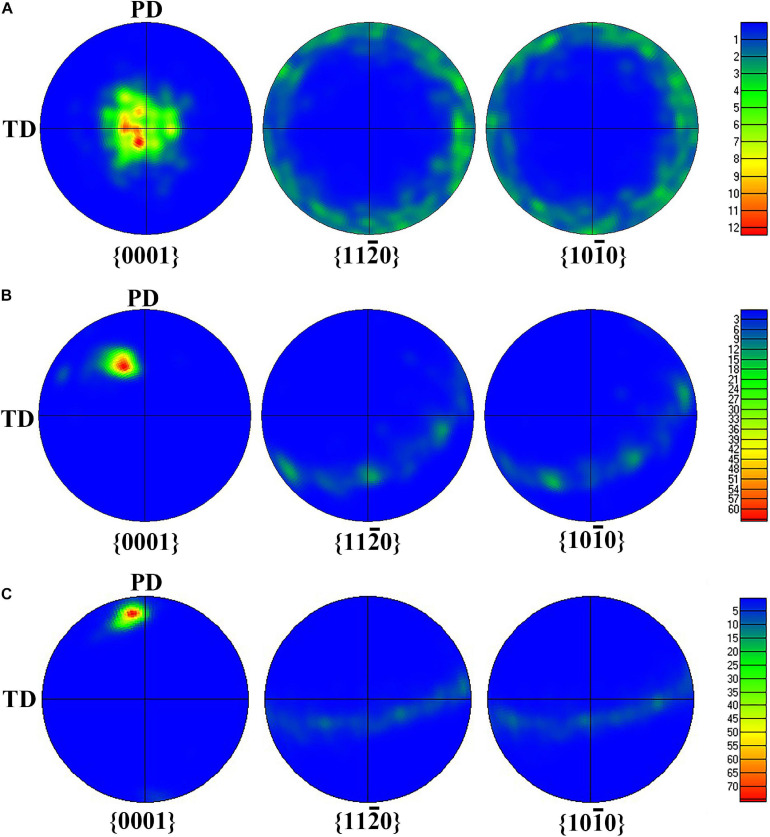
The pole figure of {0001} plane: **(A)** BM, **(B)** FSP, **(C)** FSP-ZrO_2_.

**FIGURE 6:**
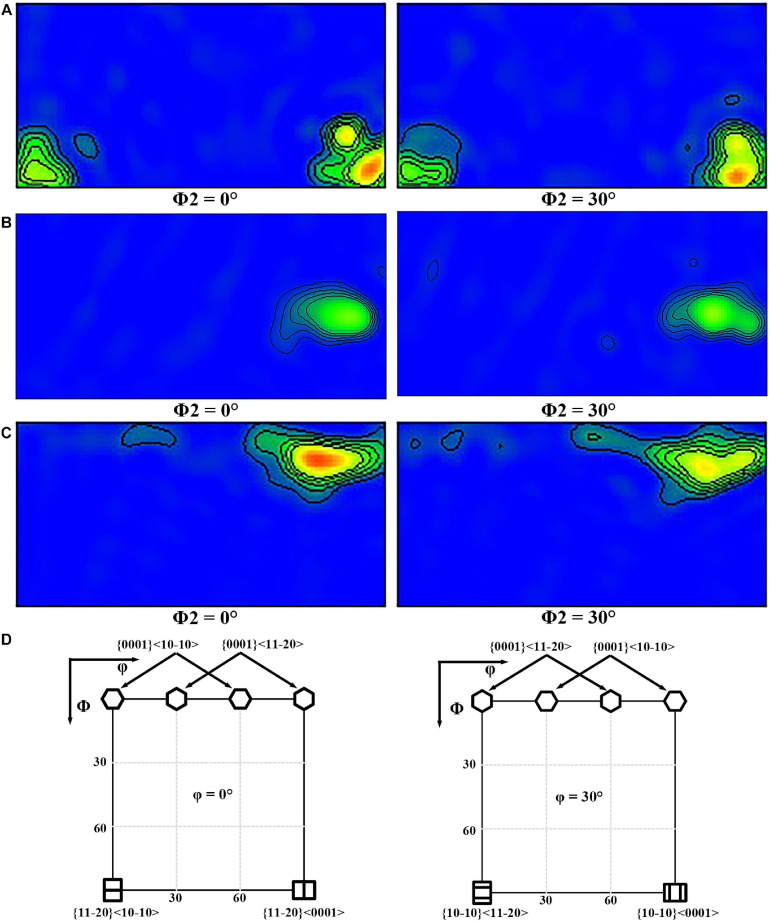
The ODF map of {0001} plane: **(A)** BM, **(B)** FSP, **(C)** FSP-ZrO2; **(D)** the standard ODF map of hcp structure.

Figures 5, 6 display {0001}, {$11\bar{2}0$}, and {$10\bar{1}0$} pole figures, and the {0001} orientation distribution function (ODF) map of BM, FSP, and FSP-ZrO_2_ samples, respectively. The c-axis of {0001} plane of BM parallel to ND (Figure 5A), showing a typical rolled texture with the polar density of 14.63, and the texture component is {$11\bar{2}0$} < 0001 > or {$10\bar{1}0$} < 0001 > texture (Figure 6A). The c-axis of {0001} plane of FSP sample is deflected from PD and TD approximately 45° and 80°, respectively (Figure 5B), and the texture component consists of {$10\bar{1}0$} or {$11\bar{2}0$} fiber texture with the polar density of 60.25 (Figure 6B). The c-axis of {0001} plane of FSP-ZrO_2_ sample is deflected from PD and TD approximately 15° and 85°, respectively (Figure 5C), and the texture component consists of {0001} < $11\bar{2}0$ or {0001} < $10\bar{1}0$ texture with the polar density of 77.21 (Figure 6C). The detailed statistical results about the texture of three samples on the {*0001*} planes are shown in Table 1. It has been reported that the deflection of c-axis on the {0001} plane of FSP sample is mainly due to the shear stress induced by shoulder and stir pin (Huang et al., 2019). It is worth noting that the deflection angle of c-axis of FSP-ZrO_2_ sample is larger than that of FSP sample, which may be due to the fact that ZrO_2_ particles increase the friction coefficient of the material during the plastic flow process, causing more grains on the {0001} plane to deflect.

**TABLE 1:** Detailed statistical results about the texture of three samples of {0001} plane.

		{0001}	
	
Samples	The angle between c-axis and TD (°)	The angle between c-axis and PD (°)	Polar density
BM	0	90	14.63
FSP	80	45	60.25
FSP-ZrO_2_	85	15	77.21

**FIGURE 7:**
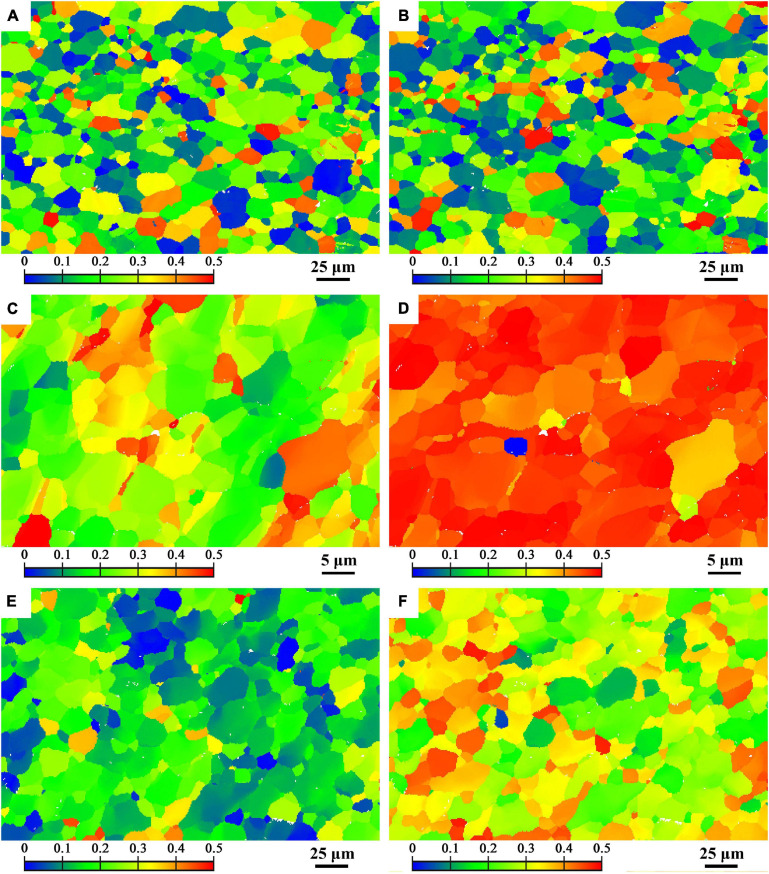
Schmid factor distribution map of TD (on the left side) and PD (on the right side) of (0001) basal slip system **(A,B)** BM, **(C,D)** FSP, **(E,F)** FSP-ZrO_2_.

Figure 7 presents the Schmidt factor (SF) distribution of (0001) basal slip along TD and PD of BM, FSP and FSP-ZrO_2_ specimens. The average SFs of BM, FSP, and FSP-ZrO_2_ specimens along TD are 0.21, 0.28, and 0.17, respectively, and the average SFs along PD are 0.21, 0.45, and 0.30, respectively. BM has the same SF value along TD and PD, and both samples after FSP have different SFs along TD and PD due to the different angles between their c-axis and TD / PD. In addition, the average SF of FSP sample is higher than that of BM, and the average SF of FSP-ZrO_2_ sample is lower than that of BM. In addition, the average SF is arranged from large to small as FSP > BM > FSP-ZrO_2_. This indicates that FSP make material soften, while ZrO_2_ particles make material harden. This phenomenon has been confirmed in the studies of Navazani and Dehghani (2016) and Jin et al. (2019).

**FIGURE 8:**
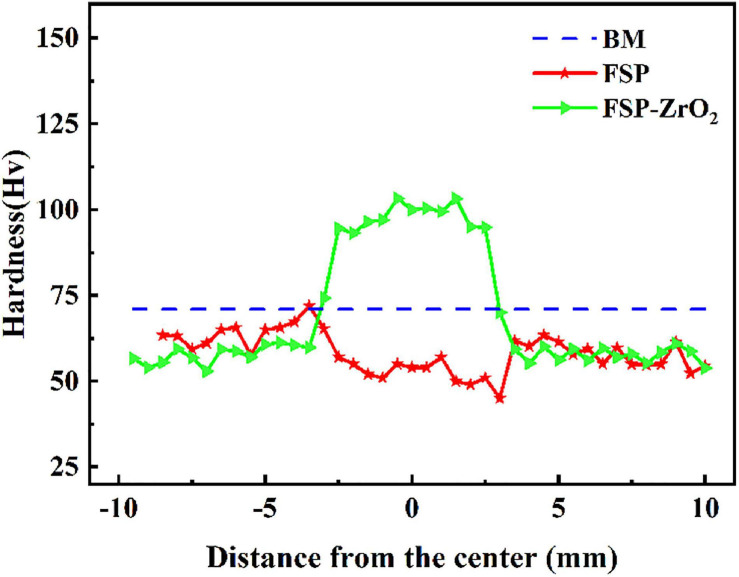
The microhardness distribution of BM, FSP and FSP-ZrO2 samples.

Figure 8 shows the microhardness distribution of the cross section of BM, FSP, and FSP-ZrO_2_ samples. The average microhardness of BM is 71 HV. The average microhardness of the FSP sample is reduced to 53 HV, which is mainly because the SF increases along PD (Figures 7C,D), causing the material to soften. The average microhardness of FSP-ZrO_2_ samples is 99 HV, which is about 40% higher than that of BM. On the one hand, ZrO_2_ particles further refine the grains of the Mg alloy during FSP (Figure 3C), and improve the strengthening effect of the refined grains. On the other hand, ZrO_2_ particles increase the resistance of dislocation movement (Vignesh et al., 2019), so that more dislocations accumulate when the material undergoes plastic deformation (Figures 3E,F), thereby strengthening the matrix.

**FIGURE 9:**
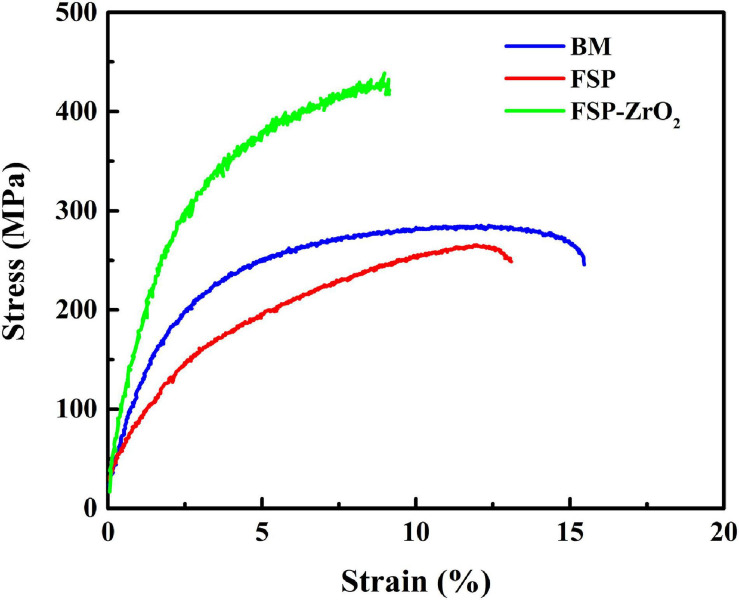
The stress-strain curves of BM, FSP and FSP-ZrO_2_ samples.

**FIGURE 10:**
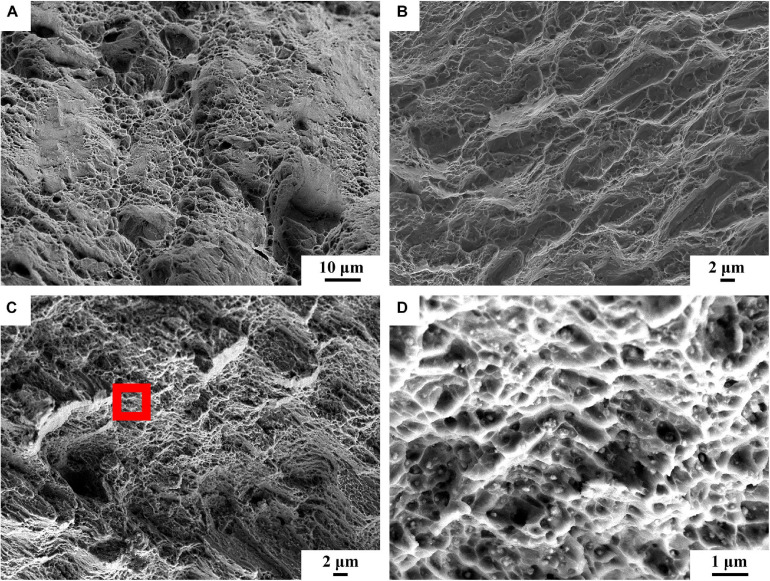
The fracture surfaces of tensile samples: **(A)** BM, **(B)** FSP, **(C)** FSP-ZrO_2_, **(D)** the enlarged view of red rectangle of **(c)**.

Figure 9 shows the stress-strain curves of BM, FSP, and FSP-ZrO_2_ samples. The ultimate tensile strength (UTS) of BM, FSP, and FSP-ZrO_2_ samples is 283, 265, and 427 MPa, the yield strength (YS) is 137, 91, and 217 MPa, and the elongation is 15.5, 13.6, and 9.5%, respectively. Compared to BM, the YS and elongation for FSP specimens reduces, and the YS of FSP-ZrO_2_ specimens increases by 80 MPa, while the elongation significantly reduces by about 6%. Since the ZrO_2_ particles impede the strain around the matrix (Figures 3E,F), thus a plastic deformation gradient is generated on the ZrO_2_/matrix interface, resulting in the uneven plasticity and the lower elongation for FSP-ZrO_2_ composites. The mechanical properties of materials are usually determined by the microstructure, and detailed analysis will be discussed later. Figure 10 shows the fracture morphology of BM, FSP, and FSP-ZrO_2_ samples after tensile testing. It can be seen that the fracture of BM is composed of dimples with different depths and cleavage surfaces (Figure 10A), showing a typical quasi-cleavage fracture. The fracture of FSP sample also presents quasi-cleavage fracture characteristics, with more uniform and fewer dimples, while more cleavage surfaces (Figure 10B), indicating a decrease in plasticity. The fracture of FSP-ZrO_2_ sample is composed of dimples and a small amount of cleavage surfaces (Figure 10C). There are a large number of ZrO_2_ particles in the dimples (Figure 10D), showing the microporous aggregate fracture characteristics. This is consistent with the previous stress-strain curve results (Figure 9).

### Electrochemical Corrosion Performance

**FIGURE 11:**
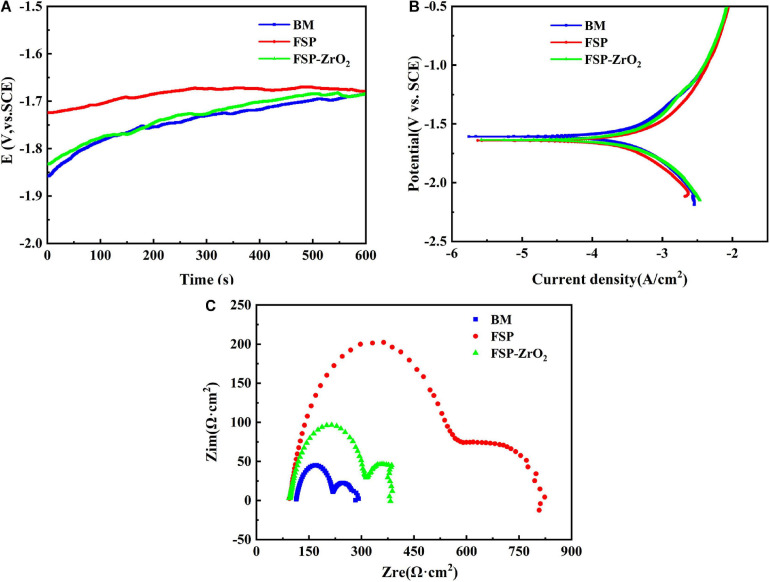
The BM, FSP and FSP-ZrO_2_ samples in SBF solution: **(A)** E versus time curves; **(B)** Tafel polarization curves; **(C)** Nyquist plots.

Figure 11A shows the curves of open circuit potential (OCP) of BM, FSP and FSP-ZrO2 samples in SBF solution with time. It can be seen that the corrosion potential of the sample increases as time increases. In the initial stage of corrosion, the OCP of BM and FSP samples are similar, while the OCP of FSP-ZrO_2_ is higher, indicating that the FSP-ZrO_2_ sample is easier to form a passivation film and has a lower self-corrosion tendency.

Figure 11B shows the potentiodynamic polarization curves of BM, FSP, and FSP-ZrO_2_ samples in SBF solution. The corrosion potential of BM and FSP samples are the same, while the corrosion potential of FSP-ZrO_2_ is lower, suggesting that FSP-ZrO_2_ samples are more sensitive to corrosion and easier to form passivation films. According to the corrosion current density, the order of samples is FSP < FSP-ZrO_2_ < BM. Corrosion rate (*P*_*i*_) that can be calculated according to Eq. (1).

(1) $${\text{P}}_{\text{i}}=22.85{\text{i}}_{\text{corr}}$$

This means that BM presents the fastest corrosion rate and FSP sample exhibits the slowest. The results of corrosion potential (*E*_*corr*_), corrosion current density (*i*_*corr*_) and corrosion rate (*P*_*i*_) of BM, FSP and FSP-ZrO_2_ samples are listed in Table 2.

**TABLE 2:** The results of *E*_*corr*_, *i*_*corr*_ and *P*_*i*_ of samples in SBF solution.

Sample	*E_*corr*_* (V)	*i_*corr*_* (mA/cm^2^)	*P_*i*_* (mm/y)
BM	−1.61 ± 0.02	0.271 ± 0.003	6.19 ± 0.02
FSP	−1.61 ± 0.01	0.090 ± 0.002	2.06 ± 0.04
FSP-ZrO_2_	−1.64 ± 0.01	0.217 ± 0.001	4.96 ± 0.02

Figure 11C exhibits the Nyquist plots of BM, FSP and FSP-ZrO_2_ samples in SBF solution. All samples are characterized by a capacitive loop in high and medium frequency range, and an inductive loop in the low frequency range. The capacitive loop is caused by the charge transfer process on the surface of the passive film, and the inductive loop is formed by the adhesion of Mg^+^ ions and Mg(OH)^+^ ions on the surface of the sample (Zhuang et al., 2015). FSP samples show the largest radius of capacitive and inductive loop, followed by FSP-ZrO_2_ samples, and finally BM. Generally, the larger the radius of capacitive and inductive loop, the better corrosion resistance of the sample (Wang et al., 2018). Furthermore, the dislocation density at the ZrO_2_ particles is too large (Figures 3E,F), and thus the local corrosion is aggravated (Liu et al., 2008). It has been reported that dislocations are easily formed between the ZrO_2_ particles and the matrix due to a large mismatch of thermal expansion coefficient between them (ZrO_2_ particles is ∼7.5 × 10^–6^ K^–1,^ Mg matrix is ∼27.1 × 10^–6^ K^–1^) (Liu et al., 2008; Vedabouriswaran and Aravindan, 2018). Therefore, the FSP-ZrO_2_ composite exhibits worse corrosion performance than FSP sample. In summary, it can be considered that the corrosion resistance of FSP samples is the best, followed by FSP-ZrO_2_, and the corrosion resistance of BM is the worst.

### SVET Voltage Maps

**FIGURE 12:**
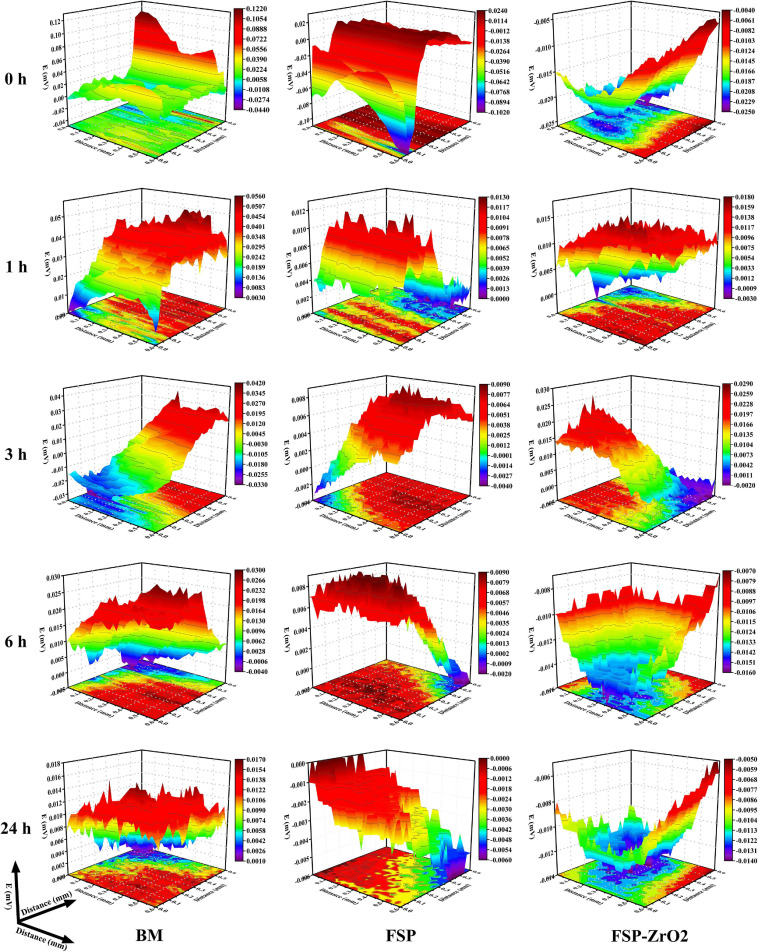
The SVET voltage maps of BM, FSP and FSP-ZrO_2_ samples in SBF solution for 0 h, 1 h, 3 h, 6 h, 24 h, respectively.

Figure 12 shows the SVET maps of BM, FSP and FSP-ZrO_2_ samples soaked in SBF at 0, 1, 3, 6, and 24 h, respectively. It can be seen that a local violent reaction occurs in BM when it is just soaked in SBF, the maximum voltage is 0.122 mv. When they are soaked for 24 h, the voltage drops to 0.017 mv. The maximum voltage for FSP samples is 0.024 mv when they are just soaked in SBF, and the voltage is reduced to 0.000 mv for 24 h. The maximum voltage for FSP-ZrO_2_ sample just immersed in SBF is −0.004 mv, and the voltage first increases and then decreases during the corrosion process. After being soaked for 24 h, the voltage drops to −0.005 mv. The above results indicate that FSP-ZrO_2_ samples are more prone to passivation during the corrosion process, followed by FSP and BM is the worst.

## Discussion

### Relationship Between Microstructure and Mechanical Property

Base metal exhibits coarse grains with inhomogeneous distribution of grain size (Figure 3A), while the FSP sample presents fine and uniform grains. During FSP, the effect of severe plastic deformation, high temperature and strain rate breaks the grains and causes dynamic recrystallization, resulting in the grain refinement of Mg alloy (Figure 3B). LAGBs entangled inside the FSP sample to form fine recrystallized grains (Figures 4A,B), indicating that continuous dynamic recrystallization (CDRX) occurs. Severe plastic deformation increases the dislocation density in grains, and the dislocations rotate or rearrange to form LAGBs in FSP process. These LAGBs gradually transform into HAGBs in the subsequent plastic deformation process (Figures 4A,B).

Compared with FSP sample, grain size of FSP-ZrO_2_ sample is refined, and HAGBs and recrystallized grains increase (Figures 3, 4). This is mainly due to the following three reasons. First, the elastic modulus of ZrO_2_ particles is different from that of Mg matrix, which will increase dislocation density and provide more nucleation sites for CDRX during FSP (Figures 3E,F). Second, ZrO_2_ particles will increase the strain and strain rate, thereby refining the grains (Mukherjee and Ghosh, 2010; Navazani and Dehghani, 2016). Third, ZrO_2_ particles pin the dislocations, hindering the movement of dislocations and growth of grain (Figures 3E,F; Mazaheri et al., 2019).

In Figures 5, 6, the grain orientation and texture of three samples have changed significantly. Mg alloy undergoes plastic flow under the combination of the shoulder and stirring pin during FSP, which greatly changes the grain orientation and texture (Yuan et al., 2011). From Figure 5B, it can be seen that the c-axis is deflected and the polar density increases for FSP sample, under the action of the compressive stress induced by the rotation of the shoulder and the shearing force of the stirring pin. For Mg alloy, basal slip has a lower critical resolved shear stress (CRSS) compared to prismatic slip and pyramidal slip (Park et al., 2003; Woo et al., 2006). Therefore, basal slip is easier to start in FSP, resulting in the formation of obvious basal texture, and the highest polar density is four times that of BM. In addition, the deflected angle of the c-axis on the {0001} plane in FSP-ZrO_2_ sample is larger than that of FSP sample, and the polar density is also significantly increased. This indicates that ZrO_2_ particles contribute to the initiation of basal slip and increase the strength of basal texture.

In general, the mechanical properties of Mg alloys are mainly affected by grain size, secondary phase and grain orientation. There is hardly precipitates in AZ31 Mg alloy, so the influence of precipitates on the alloy is not considered after FSP. During FSP, the grains of the alloy are significantly refined and uniformed, while the strength is significantly lower than that of BM (Figure 9). The grain refinement strengthening effect is not enough to compensate for the strength loss caused by other factors. Moreover, Mg alloy undergoes severe plastic deformation during FSP, and the texture composition completely different from BM is formed in the processing zone (Figures 5, 6). In addition, compared with BM, the SF value of each area for FSP sample is increased, and the basal slip is easy to proceed, so the strength of FSP sample is reduced. This reduction of strength caused by the softening of grain orientation has been confirmed in our previous studies (Wang et al., 2020). The strength of FSP-ZrO_2_ sample is significantly higher than that of BM, as shown in Figure 9. Several strengthening mechanisms of composites have been proposed (Lloyd, 1994). It has been reported that increases in the YS and UTS of FSP-ZrO_2_ composites by addition of reinforcing particles may be attributable to three factors as following (Chang et al., 2007; Navazani and Dehghani, 2016; Mazaheri et al., 2019). Firstly, the grains are obviously refined due to the dynamic recrystallization caused by FSP and the pinning effect of ZrO_2_, triggering grain refinement strengthening (Morisada et al., 2006a; Cao et al., 2014; Sun et al., 2017). Secondly, ZrO_2_ particles uniformly distributed in the matrix are pinned to the grain boundaries, hindering the movement of dislocations and causing Orowan strengthening. Thirdly, the load-transfer strengthening caused by the difference in elastic modulus between Mg matrix and ZrO_2_ particles.

Furthermore, the contribution of each strengthening mechanism to the YS of FSP-ZrO_2_ composites can be calculated using the mathematical equations as following (Hall, 1951; Zhang and Chen, 2006; Shahin et al., 2019):

(2) σy=σ0+kdy1/2-

where σ_*s*_ is the yield strength, σ_0_ is the friction stress, k_*y*_ is the stress concentration factor (5 MPa mm^1/2^), and d is the average grain size.

(3) $$\sigma_{orowan}=\sqrt{3}\frac{\text{Gb}}{\text{L}}$$

where G is the shear modulus (1.64 × 10^4^ MPa), b is Burgers vector (3.21 × 10^–10^ m), L is the average distance between ZrO_2_ particles (350 nm).

(4) $$\sigma_{LT}=\frac{\sigma_{my}f_{v}}{2}$$

where σ_*my*_ is the YS of BM (137 MPa), *f_v_* is the volume fraction of ZrO_2_ particles (about 17.6%).

Based on Eqs. (2) – (4), the calculated strength increments between the as-received AZ31 and FSP-ZrO_2_ samples were about 39.8, 26.8, and 12.1 MPa, respectively. This indicates that grain refinement strengthening contributes to improve the strength of the material, followed by Orowan strengthening, and finally load transfer strengthening. This is because the reduction of the grain size for metal matrix directly effects the increase of the strength characteristics of specimens after uniaxial tensile (Vedabouriswaran and Aravindan, 2018). Saikrishna et al. (2017) also proposed the similar conclusion. In addition, the size of the reinforcement particle (Orowan strengthening) contributes in the improvement of strength. Therefore, FSP-ZrO_2_ sample in this work presents the highest UTS and YS. Adding the contributions of various strength mechanisms, the strength increment of the material is 78.7 MPa, whereas the measured strength increment is approximately 80 MPa (Figure 9). This indicates a good agreement between the calculated and measured amounts of strength. In conclusion, the grain refinement and Orowan strengthening mechanism can be considered as the major reason of the enhanced strength for FSP-ZrO_2_ sample in this work.

### Corrosion Mechanism of the FSP-ZrO_2_

As shown in Figure 11B, BM, FSP samples and FSP-ZrO_2_ composites exhibit similar cathodic reaction and anodic dissolution characteristics. It is known that Mg and its alloys suffer electrochemical corrosion in aqueous solutions, involving cathodic reduction of water (5) and anodic dissolution of Mg (6) (Esmaily et al., 2016).

(5) $$2H_{2}\text{O}+2{\text{e}}^{-}\to {\text{H}}_{2}↑2{\text{OH}}^{-}$$

(6) $$\text{Mg}\to {\text{Mg}}^{2+}+2{\text{e}}^{-}$$

According to Eqs. (5) and (6), the overall corrosion reaction (7) during electrochemical corrosion processing as following:

(7) $$\text{Mg}+2H_{2}\text{O}=\mathrm{Mg}{\left(\mathrm{OH}\right)}_{2}+{\text{H}}_{2}↑$$

Mg dissociates in aqueous environments to form Mg hydroxide (Mg(OH)_2_) and hydrogen (H_2_) gas. The Mg(OH)_2_ corrosion film product formed on the alloy surface is a very poor electronic conductor and the rate of corrosion is hence strongly reduced (Esmaily et al., 2016).

The electrochemical corrosion test and SVET measurement of BM, FSP, and FSP-ZrO_2_ samples were carried out (Figures 11, 12) in this work. In both tests, the corrosion rate of FSP-ZrO_2_ sample is lower than that of BM (Table 2), which indicates that it has better corrosion resistance. In the electrochemical corrosion test, the corrosion resistance of FSP sample is better than that of FSP-ZrO_2_ sample, while the opposite result appears in the SVET measurement. For Mg alloys, grain size and grain orientation are the main factors affecting their corrosion resistance (Huang et al., 2019). For Mg matrix composites, in addition to the above two factors, reinforced particles also affect the corrosion resistance significantly (Vignesh et al., 2019).

Generally, grain refinement is considered to effectively improve the corrosion resistance of Mg alloys (Saikrishna et al., 2017; Wang et al., 2018; Huang et al., 2019). The corrosion resistance depends on the grain size, via the grain boundary defects. The high grain boundary energy and chemical activity promote rapid transfer rate of electrons and materials, resulting in a strong chemical reaction, so the grain boundary can provide more nucleation sites for the passive film. Fine-grained structure can provide more grain boundaries, and the smaller grain size can promote the formation of the protective Mg(OH)_2_ layer. Moreover, Choi and Kim (2015) reported that the grain boundary is conducive to stress release and can reduce the number of cracks in the protective film or corrosion layer. Therefore, grain refinement can improve the biological corrosion resistance of Mg alloy. In constant, it has been reported that the grain refinement and the increase of grain boundaries are harmful to corrosion properties. Because of the high defect density at the grain boundary, the material has a greater tendency to corrosion. According to Figures 11, 12, the grain size of FSP and FSP-ZrO_2_ samples in this study is smaller than that of BM, which means that they have better corrosion resistance, so it can be considered that grain refinement contributes to improve the corrosion resistance of FSP-ZrO_2_ composites.

On the other hand, grain orientation is also a critical factor for influencing the corrosion behavior of FSP-ZrO_2_ composites. Previous studies have shown that the (0001) plane of Mg alloy is more resistant to corrosion than (10–10) and (11–20) plane (Liu et al., 2008; Campo et al., 2014; Huang et al., 2019). The (0001) plane of Mg alloy exhibits the highest atomic density (1.13 × 10^19^, atom/m^2^) and the lowest surface energy, which leads to a low atomic dissolution rate during the corrosion. Besides, Micro-zone primary cells are formed between different grain orientations, which in turn affects the OCP (Song et al., 2010). In this study, the micro-galvanic effect in FSP and FSP-ZrO_2_ samples leads to preferential dissolution of grains with (10–10) and (11–20) orientations, resulting in the increase of OCP (Figure 11A), thus improving the corrosion resistance of the materials.

In addition, the effect of ZrO_2_ particles on the corrosion performance of FSP-ZrO_2_ sample cannot be ignored. The secondary particles improve the corrosion resistance of Mg alloy mainly in the following two ways. One is to promote grain refinement, and the other is to play a role in obstacle. According to the previous experimental results (Figures 3, 4), adding ZrO_2_ particles into AZ31 Mg with FSP can promote grain refinement, and then improve the corrosion performance. Similar results have been reported (Esmaily et al., 2016; Wang et al., 2018; Huang et al., 2019). Moreover, Vignesh et al. (2019) believed that the dispersion distribution of ZrO_2_ particles in composites increases the cumulative corrosion potential of the material, which increases the corrosion resistance of the composite. Mensah-Darkwa et al. (2013) studied the corrosion properties of Mg-hydroxyapatite (HA) composites and found that HA provides greater resistance to electrons and ions on the surface of Mg, so it essentially has greater corrosion resistance. In this study, during the corrosion process of FSP-ZrO_2_ sample, there are many dislocations (Figures 3E,F) around ZrO_2_ particles. Corrosion is more likely to occur at the position where the dislocation intersects the surface of the matrix (Liu et al., 2008). Therefore, ZrO_2_ particles can be used as obstacles to effectively prevent the penetration of corrosion and ultimately improve the corrosion resistance of the alloy. It is reported that reinforced particles can also reduce the corrosion resistance of the composites (Liu et al., 2008). For example, Saikrishna et al. (2017) prepared MWCNT/Mg composites by FSP, but its corrosion resistance was reduced because the added carbon nanotubes provide a nucleation site for galvanic corrosion. Meanwhile, this effect suppresses the effect of grain size and grain orientation on corrosion properties.

In summary, grain size, ZrO_2_ particles and grain orientation all influence the corrosion properties. Among them, the grain size is the main factor affecting the corrosion performance. According to the microstructure results, the size of FSP-ZrO_2_ (3.2 μm) is smaller than that of FSP (4 μm) and should have better corrosion resistance, which had been confirmed by SVET measurement (Figure 11). However, its electrochemical corrosion performance is lower than that of FSP sample, which may be due to the stress concentration caused by the pits left by the shedding of ZrO_2_ particles, which accelerates the corrosion rate. When the grain size is similar, ZrO_2_ particles are easy to fall off and affect the corrosion performance. In addition, grain orientation has little effect on the corrosion performance because it affects the corrosion performance by affecting the surface energy.

## Conclusion

(1) AZ31/ZrO_2_ composites with fine, densified and homogenized microstructure can be prepared by FSP. The grain size is refined from 10 μm of BM to 3.2 μm. After FSP, a strong basal texture is produced, and the c-axis of the grain is deflected under the action of compressive stress of shoulder and shear stress of pin.
(2) With the addition of ZrO_2_ particles, the microhardness of FSP-ZrO_2_ composites increases from 71 HV (BM) to 99 HV, UTS from 273 MPa (BM) to 427 MPa, YS from 137 MPa (BM) to 217 MPa. The strengthening mechanisms of the composites are mainly fine grain strengthening and Orowan strengthening. Compared with BM, the addition of ZrO_2_ particles increase the brittleness but decrease the elongation. BM and FSP samples show quasi-cleavage fracture characteristics, while FSP-ZrO_2_ composites present microporous polymerization fracture characteristics.
(3) The electrochemical corrosion behavior of three samples in SBF solution shows that the *i*_*corr*_ of FSP-ZrO_2_ composites decreases and the radius of capacitive loop increases, indicating that the corrosion rate decreases but the corrosion resistance increases for the composites. The main factors affecting the corrosion performance are grain size and ZrO_2_ particles.

## Data Availability Statement

The raw data supporting the conclusions of this article will be made available by the authors, without undue reservation.

## Author Contributions

KQ and TZ performed the experiments and wrote the manuscript. SY and SZ designed the research. ZW and PP analyzed the data. JC analyzed the data and reviewed the manuscript. LW, CL, and WW contributed to the design and data analysis. All authors read and approved the final manuscript.

## Conflict of Interest

The authors declare that the research was conducted in the absence of any commercial or financial relationships that could be construed as a potential conflict of interest.
